# Toward a more resilient Thailand: Developing a machine learning-powered forest fire warning system

**DOI:** 10.1016/j.heliyon.2024.e34021

**Published:** 2024-07-02

**Authors:** Jing Tang, Manapat Weeramongkolkul, Supanida Suwankesawong, Kumpol Saengtabtim, Natt Leelawat, Kritchart Wongwailikhit

**Affiliations:** a International School of Engineering, Faculty of Engineering, Chulalongkorn University, Phayathai, Pathumwan, Bangkok, 10330, Thailand; b Disaster and Risk Management Information Systems Research Unit, Chulalongkorn University, Phayathai, Pathumwan, Bangkok, 10330, Thailand; c Department of Industrial Engineering, Faculty of Engineering, Chulalongkorn University, Phayathai, Pathumwan, Bangkok, 10330, Thailand; d Graduate School of System Design and Management, Keio University Collaboration Complex, 4-1-1 Hiyoshi, Kohoku-ku, Yokohama, Kanagawa, 223–8526, Japan; e Department of Chemical Engineering, Faculty of Engineering, Chulalongkorn University, Phayathai, Pathumwan, 10330, Thailand

**Keywords:** Forest fire, Machine learning, Thailand, Warning system

## Abstract

Forest fires in Thailand are a recurring and formidable challenge, inflicting widespread damage and ranking among the nation's most devastating natural disasters. Most detection methods are labor-intensive, lack speed for early detection, or result in high infrastructure costs. An essential approach to mitigating this issue involves establishing an efficient forest fire warning system based on amalgamating diverse available data sources and optimized algorithms. This research endeavors to develop a binary machine-learning classifier based on Thailand's forest fire occurrences from January 2019 to October 2022 using data acquired from satellite resources, including the Google Earth engine. We use four gas variables including carbon monoxide, sulfur dioxide, nitrogen dioxide, and ozone. The study explores a range of classification models, encompassing linear classifiers, gradient-boosting classifiers, and artificial neural networks. The XGBoost model is the top-performing option across various classification evaluation metrics. The model provides the accuracy of 99.6 % and ROC-AUC score of 0.939. These findings underscore the necessity for a comprehensive forest fire warning system that integrates gas measurement sensor devices and geospatial data. A feedback mechanism is also imperative to enable model retraining post-deployment, thereby diminishing reliance on geospatial attributes. Moreover, given that decision-tree-based algorithms consistently yield superior results, future research in machine learning for forest fire prediction should prioritize these approaches.

## 1 Introduction

Forest fires have substantial and far-reaching consequences, affecting humans and natural ecosystems. These fires present immediate dangers to individuals, leading to injuries and, tragically, even death. Furthermore, the smoke contributes to air pollution, causing respiratory problems, coughing, wheezing, and eye, nose, and throat discomfort. Besides these health concerns, forest fires cause irreparable harm to the ecosystems of the affected regions, causing long-term issues such as soil erosion, biodiversity loss, and habitat destruction [1]. Additionally, fine particulate matter in smoke from forest fires (PM2.5) increases the risk of lung cancer [2]. The environmental impact is equally significant, as forest fires cause air pollution that releases carbon dioxide, a primary cause of global warming, leading to climate change and detrimental effects on plant life [3]. Moreover, the consequences of forest fires extend to ecological imbalances, resulting in heightened mortality rates among plant and animal populations and harm to their habitats [4].

Forest fires in Thailand have substantially affected the environment and economy. The World Wide Fund for Nature [5] reports that 159,490 rai were burned in 2020, equivalent to 255.184 km^2^ of land, causing damages estimated at 14,312,230,685.20 baht. The nation has grappled with persistent air pollution issues for several years, primarily attributed to agricultural burning and forest fires [6]. For example, on March 30, 2023, Chiang Mai Province recorded PM2.5 levels surpassing the World Health Organization's clean air standards by an unprecedented factor of 45.7, marking a global record [7].

**Fig. 1:**
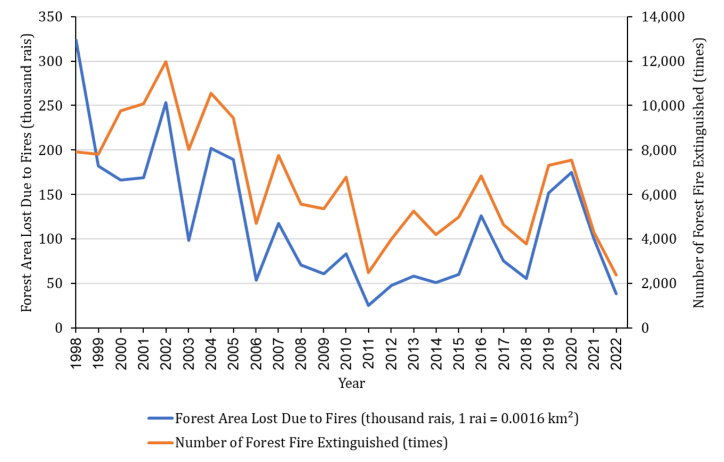
Number of forest fires extinguished, and forest area lost to forest fire in Thailand annually.

Historical data from the Forest Fire Control Office [8], a suborganization of Thailand's Department of National Parks, Wildlife, and Plant Conservation, indicates that forest fires have presented a severe challenge to the country for over 25 years. This chronic issue has necessitated the annual extinguishing of an average of 6558 forest fires, resulting in the loss of nearly 3 million rai, equivalent to 4800 km^2^ of forested land as shown in Fig. 1.

**Fig. 2:**
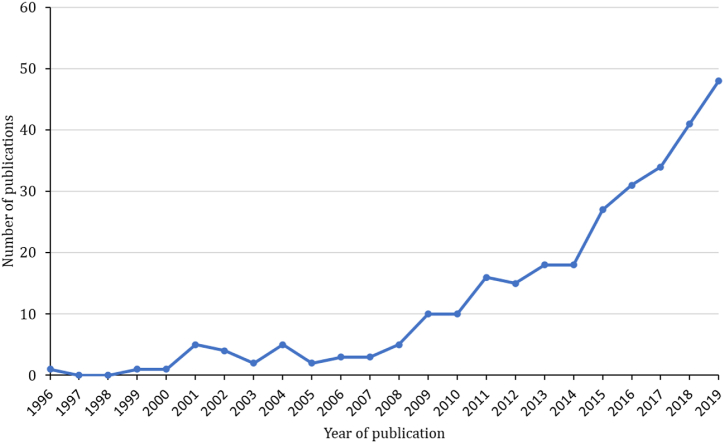
Number of publications containing the keywords “*Artificial Intelligence”* and “*Forest Fire*” over the past decades.

The Forest Fire Control Office [8] emphasizes forest fires’ intricate and multifaceted nature, necessitating a thorough investigation of the factors contributing to their initiation and spread. This exploration has generated substantial data relevant to forest fire issues. Machine learning is a subset of artificial intelligence, allowing computers to learn from data and perform tasks without explicit programming. Machine-learning models excel at automatically identifying patterns and relationships within extensive datasets. This capacity enables comprehensive data analysis, uncovering concealed insights, precise predictions, and innovative solutions to complex challenges [9]. Fig. 2 shows the exponential growth in scientific publications focusing on applying machine learning to forest fire concerns over recent decades.

However, it is imperative to emphasize that several challenges limit the application of machine learning for forest fire warnings. These challenges include the substantial infrastructure costs associated with deploying and maintaining ground-based sensors, concerns regarding data quality, the need for real-time processing, and the complexity of environmental factors influencing fire behavior. Furthermore, the scalability of machine-learning models, particularly complex deep learning models, can be challenging for large-scale monitoring. This study used various data sources to investigate the efficiency of machine-learning models focusing on forest fire prediction in Thailand. These models can be classified into three categories: linear, nonlinear, and decision-tree-based [10]. The specific models included in each category are listed in Table 1.

**Table 1:** Categories of machine-learning models and algorithms considered in this study.

Model Classification Categories	Machine-Learning Models and Algorithms
Linear Models	Logistic Regression, Naive Bayes
Nonlinear Models	K-Nearest Neighbor (KNN), Support Vector Machine (SVM), Artificial Neural Network (ANN), Voting Classifier
Decision-Tree-Based Models	Decision Tree, Random Forest, Gradient Boosting, XGBoost, LightGBM

This study uses fire radiative power (FRP) to indicate fire occurrence. FRP, as defined by the National Aeronautics and Space Administration (NASA) [11], is the rate of radiative energy emitted by a fire at the time of observation. FRP is measured in Watts using satellite technology. Bylow [12] reported that forest fires generate FRP and demonstrated that low-intensity fires typically result in insignificant FRP levels. Additionally, Kumssa [13] showed that the confidence level of fire detection increases with the increase in FRP. The FRP data employed in this study were collected from various locations in Thailand, and specific FRP values were used as forest fire occurrence indicators.

When forest fire occurred, there are multiple gases and water vapors emit to the atmosphere [14]. Urbanski et al. [15] stated that the forest fire could be the results of incomplete combustion, which lead to the release of smoke, dust, fog, ash, and various toxic gases including carbon monoxide (CO), nitrogen dioxide (NO_2_), sulfur dioxide (SO_2_), ozone (O_3_), are likely to emit to the air. Moreover, Arikan and Yildiz [16] mentioned these gases could be used to assess the quality of the air after the forest fire and environmental effects.

Recently, machine learning has been used in forest fire warning systems to deal with forest fire problems. The research can be classified into two categories based on the research objective: 1) prediction and 2) detection. Machine-learning studies have generally focused more on fire prediction than real-time detection. The objective of previous studies on forest fire prediction was to maximize the accuracy of their machine-learning models. Gulati [17] and Nissa [18] trained their models using data from the University of California, Irvine, Machine-Learning Repository. In these datasets, a confidence ratio of forest fire occurrence is included. Bui et al. [19], Amiri and Pourghasemi [20], Watson et al. [21], and Sayad et al. [22] used publicly available geographical information system data in their studies. However, a feedback system that allows machine-learning models to be retrained and model integration in real-time prediction systems have not been reported.

Another objective of previous studies on forest fire detection was to optimize the speed of the detection algorithm and warning system because the damage caused by forest fires often increases exponentially with time [23]. Unlike prediction methods, the models used in this work were integrated with sensor devices to evaluate their performance. For example, Pragati and Umbrajkar [24] combined a decision-tree model across a network of sensors to develop a fire warning system in India. However, in some studies, no further integration was made. For example, Ma et al. [25] left the integration of the model with an Internet of Things system as a future improvement.

This study aims to enhance Thailand's resilience against forest fires, a significant threat to the nation's ecosystems, economy, and environment, by developing a machine-learning-powered forest fire warning system. Existing forest fire warning systems are frequently inefficient and ineffective, requiring a more sophisticated and reliable alternative. The research question is, “How can machine learning be harnessed to create near-real-time forest fire predictions?” The hypothesis is grounded in the potential of machine-learning models to uncover complex patterns from historical data, offering a more advanced forest fire warning system. The study collects the fire warning index and potential fire occurrence indicators, uses data-preprocessing techniques to address data issues, such as missing data, disparate features across diverse datasets, and highly imbalanced data, and develops a forest fire prediction model as the foundation for intelligent forest fire warning systems. Furthermore, the research provides recommendations for these systems and outlines future developments in machine-learning solutions for forest fire prediction to reduce economic and environmental losses and improve the quality of life for forest-dependent communities in Thailand.

## 2 Method

**Fig. 3:**
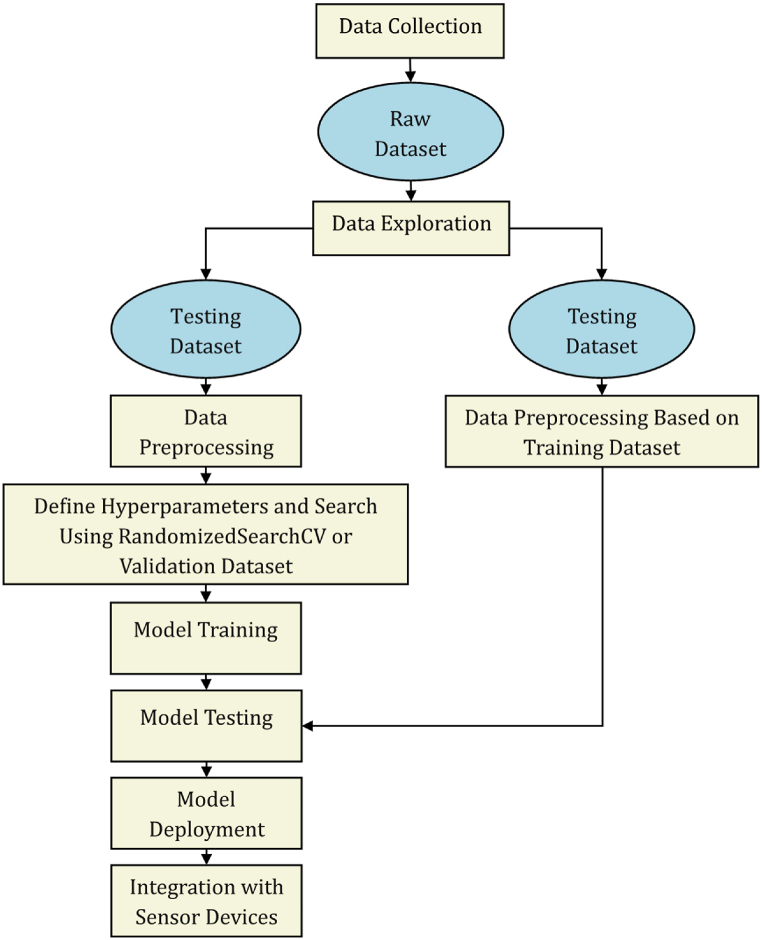
Study flowchart.

The study flowchart is presented in Fig. 3. Based on several predetermined conditions, data from multiple sources are collected and merged to form a raw dataset. After performing data exploration to understand the available data, the raw dataset is split into training and testing datasets, and the corresponding datasets are preprocessed. Randomized search cross-validation (RandomizedSearchCV) is then applied to the “cleaned” dataset to determine the best hyperparameters. Further splitting can be done to create a validation dataset that can be used for hyperparameter tuning using a similar process [9]. The testing dataset is preprocessed according to the training dataset, and the models are trained and evaluated against the testing dataset. Based on the evaluation metrics, the best model is identified and exported from the development environment to be integrated into sensor devices.

This study used Google Colab [26] as the primary integrated development environment. The Google Colab tensor processing unit (TPU) uses Cloud TPU v2 by default to accelerate the computational power. As a result, the time required for model training, particularly for complex models, is significantly reduced. Google Colab does not allow the configuration to use other versions of Cloud TPU [27]. The Python libraries and associated versions used in this study are presented in Table 2.

**Table 2:** Python libraries and their associated versions used in this study.

Library	Version
NumPy	1.22.4
Pandas	1.5.3
Matplotlib	3.7.1
Seaborn	0.12.2
Scikit-Learn	1.2.2
Imbalanced-Learn	0.10.1
XGBoost	1.7.5
LightGBM	3.3.5
TensorFlow	2.12.0
Eras	2.12.0

### 2.1 Data collection

The first step in this study was data collection. The data were collected from three distinct sources. The specific attribute analyzed was the fire warning index, which incorporates geospatial data and the atmosphere's physical properties with particular emphasis on gas data. These data were obtained from sensor devices in Thailand [28] and satellites using the Google Earth engine application programming interface (API). The API was used to process the geospatial gas data of a specified area; these data were provided by the Department of Provincial Administration of Thailand [29]. The forest fire occurrences [30], which are the points indicating where the forest fire occurred, was the dependent variable used in this study. Each of the data point location representing a district in Thailand was determined by the minimum and maximum values of the latitude and longitude of each subdistrict within a district for the gas datasets obtained from sensor devices and satellites. Furthermore, the time data of the gas dataset obtained from satellites were formatted at a period during which the satellite measured the gas values. This formatted dataset was then merged with other datasets in a subsequent step.

The data obtained from three distinct sources were combined using three different methods. Each dataset was collected over a different period; gas data were collected from sensor devices between July 21st, 2020, and November 7th, 2022, and from satellites between November 22nd, 2018, and December 14th, 2022; near real-time active-fire data, which included FRP data, were collected between January 1st, 2001 and December 17th, 2022. In the first method, gas data from sensor devices were merged with near real-time active-fire data by imposing the following conditions: the same date, the nearest hour, and the location must all fall between the maximum and minimum values of latitude and longitude for each data point. Using this method, 14 fire occurrence data points were obtained from 161,507 data points, corresponding to a rate of 0.0087 %. In the second method, satellite gas data were merged with near real-time active-fire data by imposing the following conditions: the same date, period, and location must all fall between the maximum and minimum latitude and longitude values for each data point. This method obtained 1349 fire occurrence data points from 171,893 data points, corresponding to a rate of 0.785 %. In the third method, the data collected from all three sources were merged using the condition imposed in the previous method, but no fire occurrence data points were found. The best result was obtained by merging satellite gas data with near real-time active-fire data, corresponding to the data collected between January 25th, 2019, and October 18th, 2022. This dataset was used to build the machine-learning model. Overall, there are a total of 171,893 data points with 1349 fire occurrence data for our analysis.

### 2.2 Data exploration

**Fig. 4:**
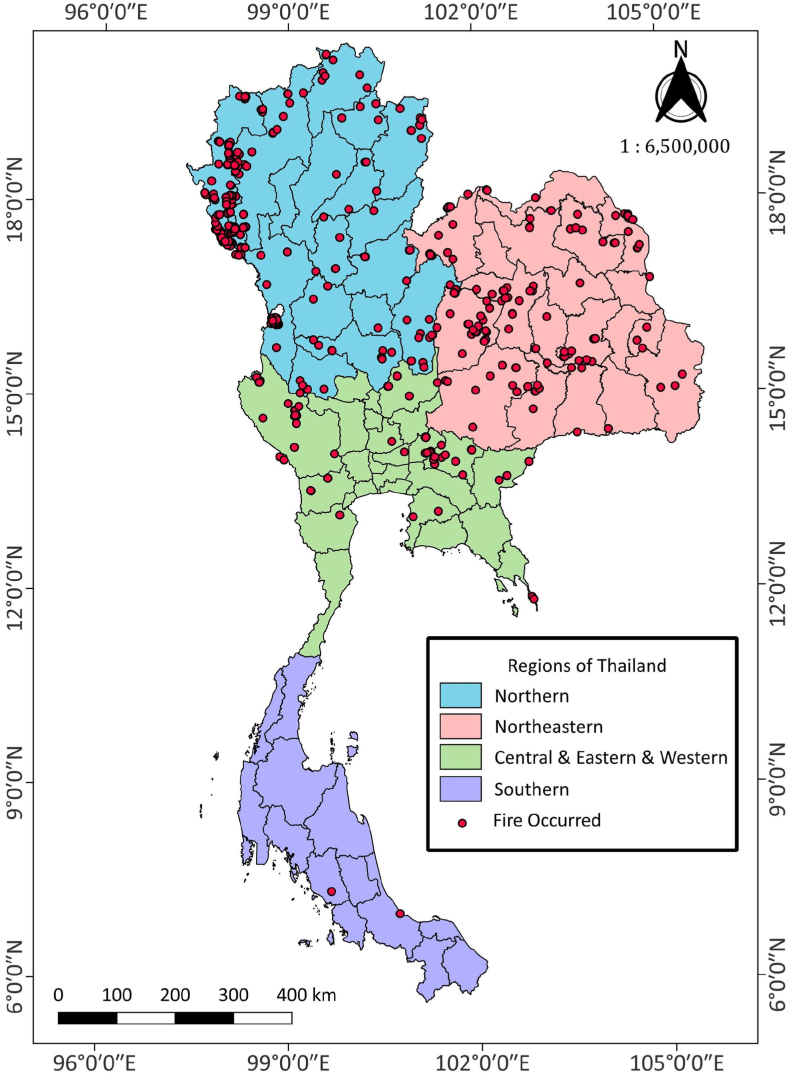
Forest fire occurrences in Thailand from 2019 to 2022 [29,30].

After data collection, some basic analysis must be performed on the collected data to understand the dataset used in the study. Overall, there are 171,893 data points which cover the area for all regions in Thailand in this research. The analysis includes (but is not limited to) the following tasks: visualizing the distribution, checking the percentage of missing values, and checking the correlation between each data attribute. Notably, some algorithms are more stringent than others regarding data preparation requirements. For example, logistic regression must avoid multicollinearity, unlike decision trees. Data exploration allows the preprocessing techniques in the subsequent step to be determined according to the requirements of each algorithm. The critical insight derived from data exploration was that the collected data are highly imbalanced, with less than 1 % of the data indicating forest fire occurrence (1: yes, 0: no). Accordingly, Fig. 4 can illustrate the forest fire occurrences based on the study area. Furthermore, in this study, there are four main gases variable that selected to classify the forest fire occurrences. These four gases include carbon monoxide (CO), sulfur dioxide (SO_2_), nitrogen dioxide (NO_2_), and ozone (O_3_). All these gases data were collected in the molar unit concentration (Mol/m^2^) format. The summarization for the descriptive statistic for these gases can be explained by Table 3.

**Table 3:** Descriptive statistic of each variable.

Variable	N	Mean	Std.Dev.	Min	Max
CO (Mol/m2)	171,893	0.255	0.104	0	0.923
NO_2_ (Mol/m2)	171,893	0.090	0.036	0	0.923
O_3_ (Mol/m2)	171,893	0.478	0.172	0	1
SO_2_ (Mol/m2)	171,893	0.196	0.043	0	1
Forest fire occurrences (Target class)	171,893	0.01	0.088	0	1

According to Fig. 4, forest fire occurrences can be represented by the red dot located in the map of Thailand. Based on Table 3, there are 171,893 data points which cover the area for all regions in the country. There are 1349 forest fire occurrences in total, which represented as a target class of this research.

Most of the forest fire occurred in the Northern Thailand. The data from the Forest Fire Control Division, Department of National Parks Wildlife and Plant Conservation, Thailand [8], allows us to understand the number of wildfire occurrence from 2019 to 2022, as summarized in Table 4.

**Table 4:** Occurrence of forest fire occurred in each region of Thailand from 2019 to 2022 [8].

Region	2019	2020	2021	2022
Northern region	5551	5435	3172	1634
Northeastern region	1047	1310	743	595
Central and Eastern region	620	765	383	138
Southern region	105	40	13	–

### 2.3 Data preprocessing

Data preprocessing allows the dataset to be prepared according to the requirements of each machine-learning model. To standardize this process for all algorithms, the features selected to be used for model training should be numeric (OneHotEncoder [9] was applied) and scaled to 0–1 (MinMaxScaler [9] was applied). The logistic regression model requires features with a high correlation among them to be dropped to avoid multicollinearity. Since the collected dataset is extremely imbalanced, imbalanced data-preprocessing techniques must be applied to the training dataset to balance its classes, thus reducing the model bias toward the majority class.

**Fig. 5:**
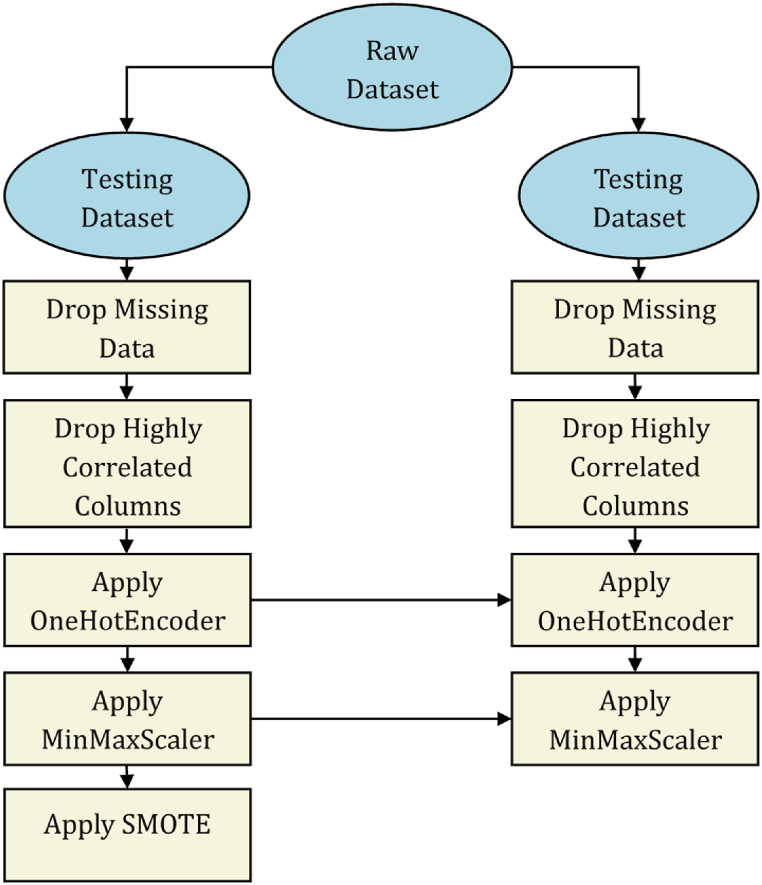
Data-preprocessing flowchart adopted in this study.

The following tasks, which are presented in Fig. 5, were performed for data preprocessing:

1. Split the raw dataset into training and testing datasets (80/20 split ratio) as appropriate. Steps 2–5 are first applied to the training dataset only.
2. Drop rows with missing features if less than 10 % of the data are missing; otherwise, drop the feature entirely.
3. Check for the correlation among features using a heat map, and drop a feature if a high correlation is identified.
4. Apply OneHotEncoder to the categorical attributes to obtain the top three categories.
5. Apply MinMaxScaler to scale all attributes to the 0–1 range.
6. Repeat steps 2–5, but preprocess the testing dataset according to the training dataset. The same OneHotEncoder and MinMaxScaler are applied to the testing dataset.
7. Apply the synthetic minority oversampling technique (SMOTE) only to the training dataset.

Due to the large size of the dataset, the common train–test split of 80:20 could be used, as recommended by Joseph [31], over other techniques such as cross-validation or the 70–30 split. Furthermore, as this study aims to determine the most appropriate machine-learning algorithms, it would be inappropriate to pre-select a specific model for cross-validation to determine the most suitable train–test split ratio as performed by Caruana and Niculescu-Mizil [32].

As for implementing imbalanced data-preprocessing techniques, it must be noted that these techniques should not be applied to the testing dataset, as they would entirely change the dataset distribution [33]. This could lead to inaccurate model performance evaluations as it may perform well on the modified testing datasets but not on new, previously unseen data. Specifically, if we apply SMOTE to the testing dataset, the model evaluation will be based on synthetic examples, creating significant misinterpretation about the model performance [34].

**Fig. 6:**
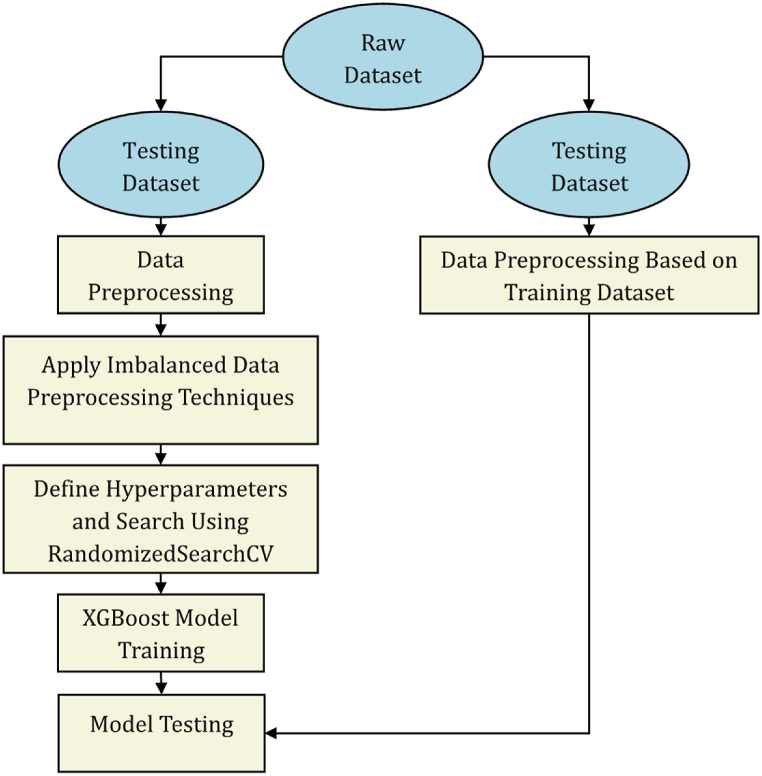
Flowchart of the imbalanced data-preprocessing evaluation adopted in this study.

Many imbalanced data-preprocessing techniques were considered in this study, including SMOTE, oversampling, undersampling, undersampling with SMOTE, and undersampling with oversampling. The process, adapted from Brownlee [35], was used to determine the most suitable preprocessing technique for imbalanced data. The step-by-step process conducted, which is presented in Fig. 6, is summarized as follows:

1. Split the raw dataset into training and testing datasets as appropriate.
2. Apply data-preprocessing steps 2–5.
3. Apply imbalanced data-preprocessing techniques to the training dataset.
4. Use RandomizedSearchCV to identify the best hyperparameters for the XGBoost models. No specific classification evaluation metric is specified for optimization.
5. Evaluate the model performance against the testing dataset to determine the most appropriate technique.

Finally, SMOTE was the best imbalanced data-preprocessing technique in all evaluation criteria except Training Time. SMOTE achieved the best Recall score, which is critical in minimizing the number of False Negative predictions in forest fires. It also achieved the best F1 score. These results indicate that SMOTE can extract the relationship between input and output variables. The evaluation results obtained using different imbalanced data-preprocessing techniques are summarized in Table 5.

**Table 5:** Evaluation results obtained using different imbalanced data-preprocessing techniques.

Imbalanced Data-Preprocessing Technique	Accuracy	Recall	F1 Score	ROC–AUC Score	Training Time
SMOTE	0.893	0.908	0.115	0.911	31 s
Oversampling	0.892	0.886	0.111	0.901	24 s
Undersampling	0.786	0.687	0.047	0.640	1 s
Undersampling with SMOTE	0.892	0.870	0.109	0.887	2 s
Undersampling with Oversampling	0.875	0.840	0.093	0.853	2 s

*Note*: The highlighted cells indicate the best performance achieved in terms of each evaluation metric.

### 2.4 Machine-learning model

After preprocessing the datasets, the machine-learning models can be trained and evaluated. The model is fed with features and label data during the training phase. Depending on the algorithm, the model can learn in various ways based on the training dataset. Once the model has been trained, it is evaluated using a separate testing dataset to evaluate its performance on the new, previously unseen data. When fed with feature data, the model prediction accuracy is compared with its prediction accuracy when fed with original label data. The optimal model and its best hyperparameters can be determined in this Model Testing phase.

#### 2.4.1 Model training

Except for Naive Bayes, Voting Classifier, and ANN, all model algorithms can be optimized by applying RandomizedSearchCV on the shortlisted hyperparameters. Naive Bayes has only one hyperparameter, “var_smoothing; ” thus, it cannot be used with RandomizedSearchCV. This parameter is used to smooth the variance of features in the training dataset. The voting classifier faces a similar problem of having only one hyperparameter called “*voting*.” This parameter specifies the type of ensemble voting used. An ANN, on the other hand, consists of hyperparameters, such as the number of hidden layers, number of neurons in each hidden layer, learning rate, activation function used in each layer, batch size, and number of epochs. These hyperparameters are difficult to evaluate with RandomizedSearchCV because the accuracy and loss curves must also be observed. Thus, a validation dataset is separated from the training dataset for hyperparameter evaluation for these three algorithms. The rest of the algorithms, such as decision tree and XGBoost, can optimize their hyperparameters using RandomizedSearchCV. As the name suggests, this technique uses cross-validation and thus does not need a validation dataset to be separated from the training dataset. The hyperparameters shortlisted for each algorithm are primarily based on the recommendations from Pedregosa et al. [9] and should be aligned with algorithms possessing similar architectural characteristics, such as XGBoost and LightGBM. These hyperparameters are listed in Table 6.

**Table 6:** Tuning hyperparameters selected for each machine-learning algorithm.

Logistic Regression	SVM	Naive Bayes	KNN	Decision Tree	Random Forest Classifier	Gradient-Boosting Classifier	XGBoost	LightGBM	ANN	Voting Classifier
Penalty	Kernel	Variance smoothing	Number of neighbors	Criterion	Number of trees	Number of boosting stages	Number of boosting stages	Number of boosting stages	Batch size	Voting
Warm start	C		Algorithm	Maximum depth	Maximum depth	Maximum depth	Maximum depth	Maximum depth	EPOCH	
Solver	Gamma			Minimum number of samples in a leaf	Maximum features	Learning rate	Learning rate	Learning rate	Number of hidden layers	
Maximum number of iterations	Class weight			Maximum number of features	Minimum number of samples in a leaf		Minimum child weight	Maximum number of leaves in one tree	Number of neurons in each hidden layer	
Dual				Splitter	Minimum number of samples required to split a node		Booster	Minimum amount of data in one leaf	Optimizer	
C									Activation function	

*Note:* Adapted from Pedregosa et al. [9].

In the RandomizedSearchCV function, the “*scoring*” parameter can be used to specify the evaluation metric, which was used to score the performance of models during the hyperparameter tuning process. Since the Recall score is considered the most important classification evaluation metric, all models that used RandomizedSearchCV are tuned to optimize the Recall score using the “*scoring*” parameter.

#### 2.4.2 Model Testing

Once the best hyperparameters for each model have been identified, the models are retrained using the training dataset and evaluated against the testing dataset in this phase. In this binary classification task, five model evaluation metrics are used: Accuracy, Recall, F1 score, ROC–AUC score (where ROC: receiver operating characteristic curve and AUC: area under the ROC curve), and Training Time. Accuracy is a classic evaluation metric in classification problems. Note that the accuracy score will be relatively high due to the highly imbalanced dataset, which includes many 0s. Thus, Accuracy is relatively less important than other evaluation metrics such as Recall. Recall indicates how many actual positive cases the model can correctly predict. It is a useful metric in cases where False Negative is of higher importance than False Positive. This metric is applicable here because the importance of a false alarm is significantly less than the importance of unpredicted forest fire occurrences. Thus, Recall is the most important evaluation metric in this study. A poor F1 score indicates underfitting in an imbalanced dataset. Although the SMOTE technique has been applied, some model algorithms can still not accurately extract the relationship between the input and output variables (even with synthetic data). Thus, the F1 score is a crucial evaluation metric in this study. The ROC–AUC score is a metric indicating how well a binary classifier can distinguish between positive and negative classes. It has a relatively low importance because it averages over all possible evaluation thresholds. Finally, the Training Time is the time required to train the final model during the “Model Testing” phase. It is used only as an additional reference, and thus, it is not a critical evaluation metric.

During the Model Testing phase, statistical tests should also be performed to ensure that the testing dataset is statistically representative of the entire dataset because the dataset is highly imbalanced. Thus, we must ensure that a statistically significant number of 1s are included in the testing dataset.

## 3 Results

The performance results obtained from the Model Testing phase are presented in Table 7.

**Table 7:** Model performance against the testing dataset for all evaluation metrics.

Model	Accuracy	Recall	F1 Score	ROC–AUC Score	Training Time
Logistic Regression	0.815	0.702	0.055	0.759	00 h 00min 06s
SVM	0.955	0.890	0.233	0.924	00 h 05min 47s
Naive Bayes	0.658	0.890	0.038	0.775	00 h 00min 01s
KNN	0.970	0.908	0.315	0.918	00 h 00min 14s
Decision Tree	0.991	0.832	0.586	0.912	00 h 00min 01s
Random Forest	0.985	0.855	0.468	0.921	00 h 05min 12s
Gradient Boosting	0.995	0.832	0.732	0.914	00 h 47min 00s
XGBoost	0.996	0.893	0.753	0.939	00 h 11min 14s
LightGBM	0.994	0.832	0.675	0.914	00 h 00min 47s
ANN	0.942	0.840	0.190	0.917	00 h 43min 22s
Voting Classifier	0.994	0.847	0.673	0.907	02 h 23min 02s

*Note*: The highlighted cells indicate the best performance achieved for each evaluation metric.

Based on the data preprocessing and machine learning model analysis, we can classify the forest fire occurrences using the four gases including CO, SO_2_, NO_2_, and O_3_. In general, many models have a high accuracy score because the data are highly imbalanced with less than 1 % being 1s. Nevertheless, the decision-tree-based models achieve the best performance in terms of accuracy. Many non-decision-tree-based models, especially linear models (i.e., logistic regression), cannot extract the nonlinear relationship between features and labels, despite employing the regularization term. The XGBoost model achieved 99.6 %, 0.753, 0.939, which represented as the best accuracy, F1 score, and ROC–AUC score, respectively compared with the other models. It also achieved the second-best Recall score, meaning that it can minimize the False Negatives to a large extent. Its Training Time is moderately long but not as long as other more complicated algorithms such as ANNs. Although the KNN model achieves the best Recall score, its low F1 score indicates that it may not be capable of extracting nonlinear relationships. Although false alarms (False Positives) are considered less important than unpredicted forest fires (False Negatives), a poor F1 score with a high Recall score indicates a very high number of False Positives in the KNN model. Thus, the best model is the XGBoost model followed by the classic gradient-boosting algorithm.

**Fig. 7:**
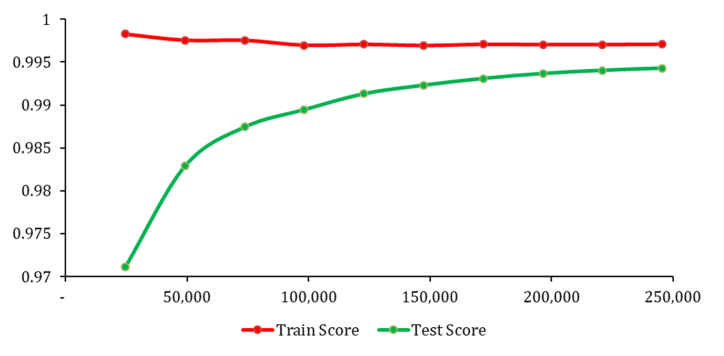
Learning curves for the optimized XGBoost model.

The training curve of the optimized XGBoost model accuracy is presented in Fig. 7. Although the training score was relatively flat, indicating that the model performance on the training set did not improve remarkably over time, the test score was curving upwards. This suggests that as the amount of training data increases, the model can generalize better to the testing dataset, despite no changes in the accuracy score of the training dataset. This phenomenon could be attributed to the application of SMOTE to the training dataset but not to the testing dataset. Nevertheless, as the test score consistently improved without any decline and considering that the testing dataset provides an accurate representation of forest fire occurrences in the real world, the performance result of this model is validated.

Although it cannot be directly adopted in a study, Table 8 shows the specific values of hyperparameters that achieved the best results. These should only be used as a reference guide in future studies to deal with similar problems.

**Table 8:** Best hyperparameter values obtained for each model.

Model	Hyperparameter	Optimal Value
Logistic Regression	Penalty	l2
	Warm start	False
	Solver	lbfgs
	Maximum number of iterations	200
	Dual	False
	C	0.1
SVM	Kernel	rcbf
	C	8
	Gamma	scale
	Class weight	balanced
Naive Bayes	Variance smoothing	1e−10
KNN	Number of neighbors	7
	Algorithm	ball_tree
Decision Tree	Criterion	log_loss
	Maximum depth	None
	Minimum number of samples in a leaf	3
	Maximum number of features	4
	Splitter	best
Random Forest	Number of trees	300
	Maximum depth	None
	Maximum number of features	2
	Minimum number of samples in a leaf	1
	Minimum number of samples required to split a node	6
Gradient Boosting	Number of boosting stages	1000
	Maximum depth	15
	Learning rate	0.2
XGBoost	Number of boosting stages	1000
	Maximum depth	10
	Learning rate	0.25
	Minimum child weight	5
	Booster	btree
LightGBM	Number of boosting stages	1000
	Maximum depth	10
	Learning rate	0.05
	Maximum number of leaves in one tree	200
	Minimum amount of data in one leaf	20
ANN	Batch size	32
	EPOCH	80
	Number of hidden layers	2
	Number of neurons in each hidden layer	10
	Optimizer	adam
	Activation function	relu, sigmoid
Voting Classifier	Voting	soft

## 4 Discussion

### 4.1 Recommendations for smart forest fire warning systems

Upon evaluation of the feature importance in the XGBoost model, it can be stated that gas measurements are important to fire prediction by the model. All gas measurement attributes are relatively equal in terms of their importance to fire prediction by the model. However, the model also highly depends on geospatial specific attributes such as latitude and longitude. When integrated into a sensor device and deployed, the geospatial attributes are constant and thus become redundant eventually. More types of data, such as temperature, humidity, and PM2.5 data, should be collected to improve the model performance in its future iterations, eventually making the model less dependent on the geospatial related attributes. However, if the data are collected from a sparse area (i.e., satellite data), the geospatial data are crucial in determining the areas with a high probability of forest fire occurrence, especially in the model first iteration. To ensure comprehensive findings, it is advisable to select attributes that cover various factors related to forest fire occurrence. By incorporating such a diverse set of attributes, the model can achieve a holistic understanding of the phenomenon, resulting in improved robustness, and accuracy in predicting forest fire events. Moreover, to mitigate potential biases in the collected data, careful attention should be given to attributes such as dates and time. It is advisable to ensure that these attributes exhibit diversity and variability by considering various intervals such as an entire day or year. By adopting such an approach, a comprehensive representation of the data can be achieved, reducing the possibility of skewed or partial outcomes. Additionally, as mentioned in the data collection step regarding the number of data points, it is highly recommended to increase the sample size. An increased sample size facilitates a more robust analysis and enhances the reliability of the findings. It also allows for a broader representation of cases and variations within the target population, ultimately leading to more accurate and reliable conclusions.

When considering the development of an alarm system after model integration, it may be useful to consider the integration of a camera module to confirm the forest fire occurrence. In this way, the model can be tuned to maximize the Recall score during the hyperparameter tuning phase. When the model predicts that a forest fire may have occurred, manual human inspection could be performed to validate the model prediction. However, a drawback of this approach is the increased cost due to the more complicated assembly of the forest fire warning system.

### 4.2 Recommendations for machine-learning pipelines

The integration of multiple data sources is considered necessary in the development of machine-learning solutions for the forest fire prediction. It is important to use multiple open-source datasets, especially in large-scale studies. This allows for multiple potentially important features to be collected. When integrating multiple data sources, it is important to take into consideration the merging columns; for example, the time window allowed must originate from the same timeframe. When multiple features are present, the first model iteration can initially utilize all these features to make its prediction. If decision-tree-based algorithms are used, it is possible to identify the model top features and use only them in future model iterations. Thus, a feedback pipeline should be developed to collect the feature data required for future model retraining. Notably, most readings indicate that forest fires do not occur (zeros). Thus, a minimum percentage threshold of forest fire occurrence, such as 1 %, should be set before loading the data through the other pipelines for preprocessing, model training, and Model Testing. Imbalanced data-preprocessing techniques should also be applied as appropriate.

An alternative solution for small-scale studies is to use one's own sensor devices to collect data from the start. However, this process may have to trade off its performance in favor of high cost and long development time required to generate the solution. Depending on the context, it may be appropriate to first start using multiple open-source datasets and slowly move toward more customized sets of features in future model iterations.

This study showed that models with linear decision boundaries do not need to be considered. These include logistic regression, which is a distance-based algorithm, and Naive Bayes, which is a probability-based algorithm. Complex models, such as ANNs, and ensemble learning algorithms, also do not necessarily improve classification performance. Instead, the focus on machine-learning research should be on decision-tree-based algorithms, including (but not limited to) decision tree, random forest, gradient boosting, XGBoost, and LightGBM.

## 5 Conclusions

In this study, a machine-learning model capable of predicting forest fires in near-real-time was developed. The model is based on forest fire data, including forest fire warning index attributes and forest fire occurrence indicators collected from datasets provided by Google Earth engine and NASA Fire Information for Resource Management System. During data preprocessing, a critical problem identified was the imbalanced distribution of the label in the datasets, leading to the evaluation of appropriate imbalanced data preprocessing techniques. The best technique identified in this study was the SMOTE technique employing the XGBoost algorithm as a base. On this basis, various models were trained, and evaluated under the code pipelines, demonstrating that the XGBoost algorithm provides the most optimal result based on the classification evaluation metrics defined.

The findings indicated that the integration of machine-learning techniques with forest fire sensors can significantly enhance the reliability and efficiency of fire warning and monitoring systems. Machine learning algorithms can analyze large datasets of sensor data, detect patterns and anomalies, and accurately identify fires in a timely manner. This enables fire managers and emergency responders to quickly respond, thus reducing the risk of property damage and loss of life. Consequently, the main advantage of using machine-learning techniques in conjunction with sensors is the improved accuracy and efficiency of fire detection and monitoring systems.

### 5.1 Contribution

The use of machine-learning algorithms in forest fire prediction is a significant contribution that can be applied globally, not only in Thailand but also in other countries facing forest fires, including Chile, Canada, Greece, Portugal, Spain, and the United States. Forest fire management organizations worldwide, such as the National Park, Wildlife, and Plant Conservation of Thailand, as well as other international organizations related to forest fires, such as the United Nations Framework Convention on Climate Change and the International Association of Wildland Fire, can benefit from the results of this study.

In addition, this study also indicated that future research on forest fire prediction using machine-learning techniques should concentrate on decision-tree-based algorithms, as they outperform other machine-learning algorithms in terms of many evaluation metrics. This could further advance the research field of forest fire prediction.

### 5.2 Limitation

This study has also indicated some limitations, which must be taken into consideration. First, the dataset employed may be inadequate due to the low percentage of fire occurrences used to train the model, resulting in an imbalanced dataset that requires the SMOTE technique to effectively increase the number of data indicating forest fire occurrences. Additionally, the data were obtained from various sources; integrating them into a single dataset may introduce errors because of the specified conditions employed to merge the different datasets, such as using a range of latitude and longitude to combine two datasets. This leads to an area being represented as a rectangle rather than its exact shape. This approach may cause inaccuracies in the location of real data and thus adversely affect the model performance.

### 5.3 Future study

In a future study, the focus should be on the data collection process, as this is a key limitation of this study. If possible, direct on-site data collection should be performed to make the model more suitable for the area where it will be deployed. This study has also demonstrated that decision-tree-based algorithms should focus on model development, meaning that researchers could spend more time in other areas of model development such as data collection planning.

## Data availability statement

This study uses the data from three sources: (1) gas data from Ref. [28], (2) LAT/LONG coordinates indicating the name of the sub-district, district, and province data from Ref. [29], and (3) FRP data from Ref. [30].

## CRediT authorship contribution statement

**Jing Tang:** Writing – review & editing, Writing – original draft, Supervision, Methodology, Investigation, Conceptualization. **Manapat Weeramongkolkul:** Writing – review & editing, Writing – original draft, Methodology, Formal analysis, Data curation. **Supanida Suwankesawong:** Writing – review & editing, Writing – original draft, Formal analysis, Data curation. **Kumpol Saengtabtim:** Writing – review & editing, Writing – original draft, Supervision. **Natt Leelawat:** Writing – review & editing, Writing – original draft, Supervision, Project administration, Methodology, Investigation, Funding acquisition, Conceptualization. **Kritchart Wongwailikhit:** Data curation.

## Declaration of competing interest

The authors declare that they have no known competing financial interests or personal relationships that could have appeared to influence the work reported in this paper.
